# West Sweden Asthma Study: Prevalence trends over the last 18 years argues no recent increase in asthma

**DOI:** 10.1186/1465-9921-10-94

**Published:** 2009-10-12

**Authors:** Jan Lötvall, Linda Ekerljung, Erik P Rönmark, Göran Wennergren, Anders Lindén, Eva Rönmark, Kjell Torén, Bo Lundbäck

**Affiliations:** 1 Department of Internal Medicine, Krefting Research Centre, Sahlgrenska Academy, University of Gothenburg, Gothenburg, Sweden; 2 Department of Pediatrics, Sahlgrenska Academy, University of Gothenburg, Gothenburg, Sweden; 3 Department of Environmental & Occupational Medicine, Sahlgrenska Academy, University of Gothenburg, Gothenburg, Sweden; 4 The OLIN Studies, Department of Medicine, Sunderby Central Hospital of Norrbotten, Luleå, Sweden; 5 Environmental & Occupational Medicine, Department of Public Health and Clinical Medicine, University of Umeå, Umeå, Sweden

## Abstract

Asthma prevalence has increased over the last fifty years, but the more recent changes have not been conclusively determined. Studies in children indicate that a plateau in the prevalence of asthma may have been reached, but this has not yet been confirmed in adults. Epidemiological studies have suggested that the prevalence of asthma in adults is approximately 7-10% in different parts of the western world.

We have now performed a large-scale epidemiological evaluation of the prevalence of asthma and respiratory symptoms in adults between the ages of 16-75 in West Sweden. Thirty thousand randomly chosen individuals were sent a detailed questionnaire focusing on asthma and respiratory symptoms, as well possible risk factors. Sixty-two percent of the contacted individuals responded to the questionnaire. Asthma prevalence, defined as asthma diagnosed by a physician, was 8.3%. Moreover, the prevalence of respiratory symptoms was lower compared to previous studies. The most common respiratory symptom was any wheeze (16.6%) followed by sputum production (13.3%). In comparison with studies performed 18 years ago, the prevalence of asthma has not increased, and the prevalence of most respiratory symptoms has decreased. Therefore, our data argues that the continued increase in asthma prevalence that has been observed over the last half century is over.

## Introduction

In terms of prevalence and morbidity, asthma has increased in most parts of the world during the second half of the past century [1-3]. The increase was first recognised in Australia, New Zealand and in areas of the United Kingdom (UK) and the USA, countries in which the mortality in asthma also increased at the time [1,4,5]. Less change in the prevalence, morbidity and mortality was seen in Continental [6,7] and Eastern Europe [8]. In Eastern Europe, different diagnostic traditions compared to Western Europe partly explained a lower prevalence [9,10]. During the last decades of the century a marked increase in asthma was also detected in developing countries [11], particularly in large cities [7,11], while the prevalence did not change considerably in rural areas of Africa and China [12,13]. Recent studies, including the ISAAC III [14], suggest that the increase in asthma among children and adolescents has leveled off in several westernized countries [15-17]. However, in some of these countries, such as Germany and UK, studies also point out diverging and opposite trends [17-19]. In contrast to children, there are no recent published studies of the change of prevalence in adult asthma and symptoms common in asthma.

In Sweden the asthma prevalence increased from 2-3% in the 1970s [20,21], to approximately 5% in the 1980s [22,23] and to 8-10% in the mid 1990s [24-26]. The increase was first noticed in the north of Sweden [22,27] and was, to some extent, explained by an increase in diagnostic activity [28]. Notably, there still seems to be a north-south gradient in the prevalence of asthma with a slightly higher prevalence in the north [24]. Population based data of asthma prevalence among adults in Sweden has not been published for the past ten years.

In 2008, a large study focusing on asthma and allergic diseases was initiated in West Sweden. The first part of the study was a postal questionnaire survey on respiratory symptoms and diseases, as well as allergic conditions. The initial aim was to update the prevalence of asthma, allergy and respiratory symptoms, which is presented here. Our hypothesis was that the increasing trend of adult asthma in Sweden has reached a plateau.

## Materials and methods

### Study area and population

**Figure 1:**
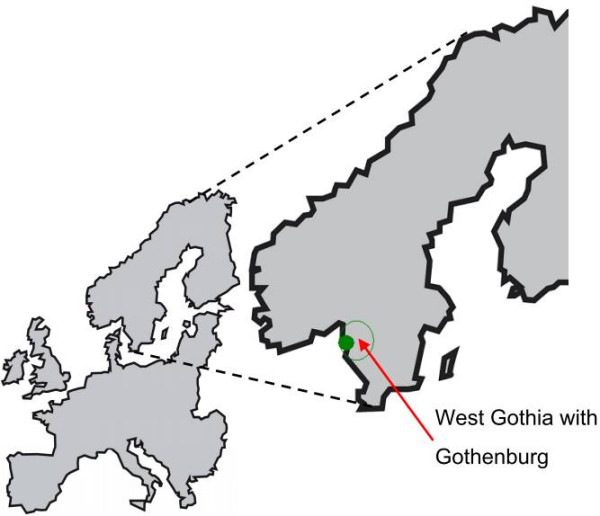
**Sweden with the city of Gothenburg and the region of West Gothia**.

The study area is the region of West Sweden (Figure 1), with the city of Gothenburg located at the North Sea. Gothenburg is the second largest city in Sweden and had a population of 494 000 at the end of 2007, with more than 700 000 when including the whole urbanised area surrounding the city. The population of the West Gothia region was 1 547 000 in 2007, which corresponds to approximately 1/6 of the Swedish population. A postal questionnaire was mailed in February of 2008 to 30 000 inhabitants in the region, aged 16-75. A random selection of 15 000 subjects was chosen from the population living in the urbanised area of Gothenburg and its surrounds. Similarly a random sample of 15 000 subjects of the same age was chosen from the rest of the West Gothia region. The names and addresses were received from the Swedish Population Register.

### Methods

External companies administered the questionnaires with cover letters and prepaid envelopes for returning the completed questionnaires, as well as the computerisation of collected data. Non-responders received three reminders. The invited individuals were also given the option to respond over the internet.

The questionnaire included three parts. The first part was a modified version [29] of the Swedish OLIN study questionnaire [23] that has been used in several studies in northern Europe [24-27] and contained questions about asthma, rhinitis, chronic bronchitis/COPD/emphysema, respiratory symptoms, use of asthma medication and possible determinants of disease, such as smoking habits and family history of airway diseases. The second part included questions about occupation, airborne occupational and environmental exposures, socio-economic status and health status. The third part consisted of the Swedish version of the GA^2^LEN questionnaire, which added detailed questions about rhinitis and eczema.

### Definitions

#### Ever asthma

"Have you ever had asthma"; *Physician-diagnosed asthma*: "Have you been diagnosed as having asthma by a doctor"; *Active asthma*: Reported ever asthma or physician diagnosed asthma *and *at least one out of: use of asthma medicine, attacks of shortness of breath, any wheeze, or recurrent wheeze; *Use of asthma medicine*: "Do you currently use asthma medicine (permanently or as needed)"; *Rhinitis*: "Have you been diagnosed as having allergic rhinitis/hay fever by a doctor".

#### Attacks of shortness of breath

"Do you presently have, or have you had in the last 10 years, asthma symptoms (intermittent breathlessness or attacks of shortness of breath; the symptoms may exist simultaneously with or without cough or wheezing)" *and *"Have you had these symptoms within the last year".

#### Any wheeze

"Have you had whistling or wheezing in the chest at any occasion during the last 12 months"; *Wheezing with breathlessness*: Yes to *any wheeze *and "Have you been at all breathless when you had wheezing or whistling in the chest"; *Wheezing apart from cold*: Yes to *any wheeze *and "Have you had this wheezing or whistling in your chest when you have not had a cold"; *Wheezing with breathlessness apart from cold*: Yes to *any wheeze *and "Have you been at all breathless when you had wheezing or whistling in the chest" and "Have you had this wheezing or whistling in you chest when you have not had a cold"; *Recurrent wheeze*: "Do you usually have wheezing or whistling in your chest when breathing";

#### Longstanding cough

"Have you had a persisting cough during the last year"; *Sputum production*: "Do you usually have phlegm when coughing or do you have phlegm in the chest which is difficult to bring up"; *Chronic productive cough: Sputum production *for at least three months during two subsequent years.

*Smokers *reported smoking during the year preceding the survey; *Ex-smokers *reported having quit smoking at least 12 months preceding the survey; *Non-smokers *reported neither smoking nor ex-smoking.

An evaluation by telephone interviews showed no statistically significant differences regarding asthma and symptom prevalence between those that responded to the postal survey and those that did not. Furthermore, the number of reminders sent out did not change the overall results of the study [30].

### Comparison with previously performed studies

The results of the current survey were compared with the results from two previous studies in defined geographic areas within the region of West Gothia. The first study, the Gothenburg part of the European Community Respiratory Health Survey (ECRHS), was performed on the island of Hisingen in the city of Gothenburg. This study was performed in 1990, with 2884 participants aged 20-44 years [31]. The second study was conducted in 1994 in a southern part of the West Gothia region, the former county of Southern Älvsborg, with 15 813 participants aged 16-50 [32]. In order to compare results, subsets of the current study from the same areas and of the same age compositions were used. These subsets in each of the two areas consisted of 1238 and 1167 subjects respectively. The comparisons were based on the results of identical or very similar questions used in the comparison studies.

### Ethical approval

The study was approved by the Ethics Committee at the University of Gothenburg.

### Analyses

Ten percent of the data was entered twice for quality control of the computerisation. Errors amounted to 0.1-0.2% of the computerised data with only a few exceptions. Statistical analyses were performed using SPSS version 16.0. Comparisons of proportions were tested with a chi-square test or Fisher's exact test and comparisons of means were tested with a two-tailed Student's t-test. One way analysis of variance (ANOVA) was used for test for trends. A p-value of < 0.05 was regarded as statistically significant. Covariates used in the analyses included age, sex, family history of asthma, smoking habits, area of domicile and rhinitis. Rhinitis was used as a surrogate variable for atopy. Multiple logistic regression models were performed using these independent variables as risk factors (odd ratios (OR), with 95% confidence intervals (CI)) of asthma and respiratory symptoms.

## Results

### Participation and smoking

Of the 30 000 subjects randomly selected for the questionnaire, at least 782 were not traceable, resulting in an actual study sample of 29 218 subjects, of which 18 087 (62%) participated. Only 814 subjects, 4.5% of the responders, used the option to answer over the internet. Female sex and domicile outside of the metropolitan area was significantly associated with being traceable (Table 1). Women had a higher response rate (67%) compared to men (56%, p < 0.001). There was a greater response rate among participants living outside the metropolitan area of Gothenburg compared to within (64% vs. 60% respectively; p < 0.001). Participation increased significantly by age (p < 0.001), from 51% among those aged 16-25 years to 77% among the oldest aged 66-75 years (Table 1).

**Table 1:** Study population by age, sex and area.

		Age (years)						Sex			Area			
Study population		16-25	26-35	36-45	46-55	56-65	66-76	Men	Women	p-value	West Gothia	Gothenburg	p-value	Total
**Initial study sample**	N													**30000**
Not possible to trace	N (%)	157 (2.3)	165 (2.8)	80 (1.4)	49 (1.0)	26 (0.5)	12 (0.4)	311 (2.0)	178 (1.2)	< 0.001	175 (1.2)	314 (2.1)	< 0.001	489 (1.6)
Deceased	N (%)	1 (0.02)	1 (0.02)	1 (0.02)	3 (0.06)	2 (0.04)	9 (0.3)	11 (0.07)	6 (0.04)	0.225	9 (0.1)	8 (0.1)	< 0.001	17 (0.06)
Moved	N (%)	40 (0.7)	24 (0.4)	12 (0.2)	3 (0.06)	2 (0.04)	6 (0.2)	41 (0.3)	46 (0.3)	0.594	30 (0.2)	57 (0.4)	0.005	87 (0.3)
Not able due to disease or disability	N (%)	11 (0.2)	11 (0.2)	14 (0.2)	18 (0.4)	33 (0.7)	34 (1.2)	66 (0.4)	55 (0.4)	0.318	64 (0.4)	57 (0.4)	0.525	121 (0.4)
Other causes	N (%)	11 (0.2)	14 (0.2)	9 (0.2)	13 (0.3)	9 (0.2)	12 (0.4)	37 (0.3)	31 (0.2)	0.544	32 (0.2)	36 (0.2)	0.716	68 (0.2)
														
**Real study sample**	N	**5242**	**5653**	**5593**	**4947**	**4941**	**2842**	**14534**	**14684**		**14691**	**14527**		**29218**
Did not want to participate or returned a blank questionnaire	N (%)	61 (1)	50 (1)	56 (1)	62 (1)	88 (2)	82 (3)	186 (1)	213 (2)	0.226	222 (2)	177 (1)	0.034	399 (1)
Non-responders	N (%)	2577 (49)	2484 (44)	2247 (40)	1660 (34)	1194 (24)	570 (20)	6158 (42)	4574 (31)	< 0.001	5039 (34)	5693 (39)	< 0.001	10732 (37)
**Responders**	N (%)	**2604 (51)**	**3119 (55)**	**3290 (59)**	**3225 (65)**	**3659 (74)**	**2190 (77)**	**8190 (56)**	**9897 (67)**	< 0.001	**9430 (64)**	**8657 (60)**	** < 0.001**	**18087 (62)**

Difference (p-value) by sex and area.

The prevalence of smoking was highest among women (20%) compared to men (18%) (p = 0.001) overall and in most age groups (Table 2), while being both ex-smokers and non/never smokers were similarly common in men and women. There were no major differences in the prevalence of smoking by age group, except in the age group of 66-75 years, of which 14% were smokers.

**Table 2:** Smoking habits (%) by age and sex.

	Age (years)												Sex		Area	
Smoking status	16-25 years		26-35 years		36-45 years		46-55 years		56-65 years		66-76 years					
	Men	Women	Men	Women	Men	Women	Men	Women	Men	Women	Men	Women	All men	All women	Gothenburg	West Gothia
Non-smokers	79.4	69.4	71.2	68.2	66.2	59.8	52	46.5	43.3	47.8	46.2	57.4	59	58	58	59
Ex-smoker	3.9	6.9	12.2	15.6	16.4	19.9	27.3	29.8	36.7	31.6	37.7	28.8	23	22	22	23
Smokers	15.8	23.4	16.2	15.9	16.9	19.7	20	23.2	19.4	19.9	15.7	13.3	18	20	20	17

### Prevalence - asthma

The prevalence of *physician-diagnosed asthma *was 8.3% (women 9.1%; men 7.4%, p < 0.001). Asthma prevalence was 9.6% in 16-25 year old subjects, which increased to 10.2% in those aged 26-35 years, and then decreased significantly by increasing age to 7.1% in 66-75 year old subjects (Table 3). *Ever having asthma *was reported by 9.7% (women 10.5%; men 8.7%, p < 0.001) and the prevalence of having either *physician-diagnosed asthma *or *ever asthma *was 10.2%. The use of *asthma medicines *was reported by 6.8% of men and 10.1% of women (p < 0.001). Of those reporting physician-diagnosed asthma, 70% reported using asthma medication.

**Table 3:** Prevalence (%) by age, sex and area.

		Age (years)							Gender				Area		
Symptom or disease		16-25	26-35	36-45	46-55	56-65	66-75	test for trend	M	W	p-value	Total	Gothen-burg	West Gothia	p-value
**Ever asthma**		11.2	12.2	10.2	8.5	8.2	7.6	< 0.001	8.7	10.5	< 0.001	9.7	9.9	9.5	0.330
**Physician-diagnosed asthma**		9.6	10.2	8.4	7.2	7.4	7.1	< 0.001	7.4	9.1	< 0.001	8.3	8.4	8.3	0.759
**Asthma medicine**		9.3	8.9	8.3	8.3	8.2	8.9	0.293	6.8	10.1	< 0.001	8.6	8.7	8.5	0.780
**Rhinitis**		27.8	33.5	31.5	27.4	22.4	16.1	< 0.001	26.0	27.6	0.020	26.9	28.3	25.6	< 0.001
															
**Attacks of SOB**		9.3	9.9	9.8	9.3	9.0	10.0	0.764	7.5	11.2	< 0.001	9.5	9.9	9.1	0.066
**Recurrent wheeze**		5.0	5.4	6.3	7.8	7.9	8.8	< 0.001	6.8	6.9	0.976	6.8	7.2	6.5	0.056
**Any wheeze**		16.0	16.9	16.3	17.2	16.7	16.1	0.850	15.3	17.6	< 0.001	16.6	17.3	15.9	0.014
**Wheeze with breathlessness**		10.1	10.9	10.8	11.3	10.4	9.7	0.633	9.1	11.8	< 0.001	10.6	11.1	10.1	0.030
**Wheeze without cold**		8.6	9.9	8.9	10.2	9.7	9.8	0.137	8.9	10.1	0.008	9.5	10.1	9.0	0.012
**Wheezing with breathlessness apart from cold**		5.8	6.3	5.9	6.2	5.7	5.8	0.678	5.3	6.4	0.002	5.9	6.4	5.5	0.013
															
**Longstanding cough**		13.3	11.3	11.1	10.7	11.3	11.3	0.056	10.2	12.4	< 0.001	11.4	12.1	10.8	0.004
**Sputum production**		15.6	13.0	12.1	12.0	12.8	15.7	0.617	13.1	13.5	0.429	13.3	14.4	12.3	< 0.001
**Chronic productive cough**		4.0	5.0	5.4	6.7	6.8	9.0	< 0.001	6.1	6.1	1.000	6.1	6.4	5.7	0.043
															
**Dyspnoea**		3.5	3.2	4.5	6.4	9.9	12.9	< 0.001	4.8	8.1	< 0.001	6.6	6.7	6.5	0.611

Difference (p-value) by age, sex and area.

**Figure 2:**
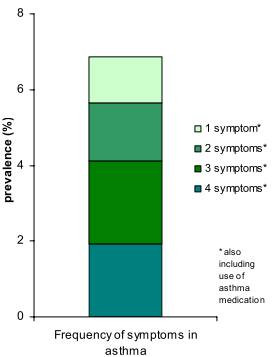
**Prevalence of active asthma in relation to the number of symptoms or use of asthma medicine**.

*Active asthma *(ever having asthma and having symptoms or using asthma mediation) was detected in 6.9% of the sample. Of these, 84.2% reported use of *asthma medication*, 73.0% *attacks of shortness of breath*, 39.1% *any wheeze *and 74.1% *recurrent wheeze*. The number of symptoms and/or use of asthma medicines among subjects with active asthma are shown in Figure 2. Forty-six percent of the 5.9% reporting *wheezing with breathlessness apart from cold *had not reported they had *ever asthma *or *physician-diagnosed asthma*, a result corresponding to 2.7% of the participating study sample.

### Prevalence - respiratory symptoms

The most common respiratory symptom reported was *any wheeze *(16.6%), followed by *sputum production *(13.3%), *longstanding cough *(11.4%) and *attacks of shortness of breath *(9.5%). *Recurrent wheeze *was reported by 6.8% and *chronic productive cough *by 6.1%, with both equally common in men and women. Most symptoms common in asthma were significantly more prevalent among women, while bronchitic symptoms were equally common in men and women. *Ever asthma*, *physician-diagnosed asthma *and *use of asthma medicines *were equally common in the metropolitan area of Gothenburg and the remaining part of West Gothia, while most symptoms were slightly but significantly more common in Gothenburg. Prevalence of symptoms by age, gender and domicile area is reported in Table 3.

### Comparison with previous studies

**Figure 3:**
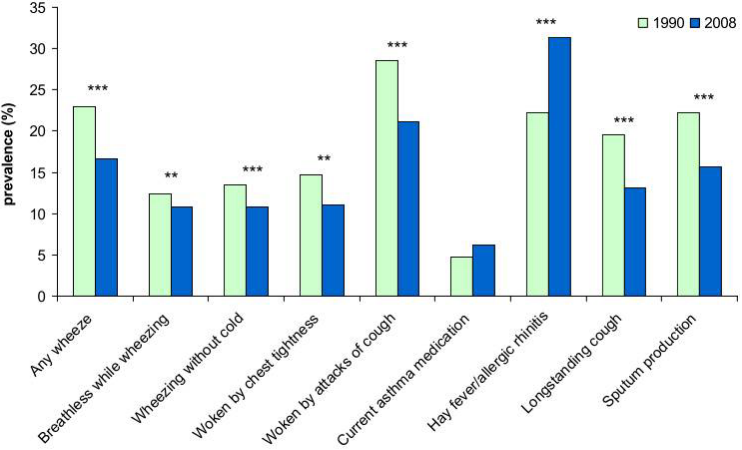
Comparison of the prevalence of respiratory symptoms using identical questions in the ECRHS Study (1990) and in the current study (2008) among 20-44 year old subjects living in the area of Hisingen, Gothenburg (* p < 0.05, ** p < 0.01, ***p < 0.001).

When comparing the results of the current study with the ECRHS study performed in 1990 [31], the prevalence of most airway symptoms had decreased considerably and significantly between 1990 and 2008. Specifically, *any wheeze *had been reduced from 23% to 17% (p < 0.001), *sputum production *from 21% to 15% (p < 0.001) and *longstanding cough *from 18% to 12% (p < 0.001), while reports of asthma indicate some increase asthma from 6% to 8%, and a slightly increased use of asthma medicines from 5% to 6% (Figure 3). The decrease in symptom prevalence was accompanied by a 50% reduction in smoking prevalence from 42% to 21%.

Comparisons were also made with a study performed in the former county of Southern Älvsborg in the southern part of the region in 1994 [32]. While *any wheeze *decreased from 18.2% to 15% (p < 0.001) and *attacks of shortness of breath *did not change (7% vs. 8%), *sputum production *increased from 9% to 11% (p = 0.015). All outcomes relating to asthma increased significantly, including *ever asthma *from 6% to 11% (p < 0.001), and both *physician-diagnosed asthma *as well as *use of asthma medicines *increased from 5% to 9% (p < 0.001). The prevalence of smoking decreased over these 14 years from 32% to 18% (p < 0.001).

### Multivariate relationships - risk factors for asthma and symptoms

In the risk factor analyses using multiple logistic regression, the dependent variables include *physician-diagnosed asthma*, *attacks of shortness of breath*, *any wheeze*, *recurrent wheeze *and *sputum production*. For *physician-diagnosed asthma*, rhinitis was the dominant risk factor yielding an OR of 5.41 (95% CI 4.81-6.08) followed by family history of asthma, OR 2.61 (2.31-2.94). Female sex was significantly associated with physician-diagnosed asthma; OR 1.17 (1.05-1.32), as was ex-smoking; OR 1.34 (1.16-1.55) while current smoking was borderline significant. Having an age of 16-30 resulted in the highest risk of having asthma (Table 4).

**Table 4:** Risk factors for asthma and respiratory symptoms by multiple logistic regression analysis.

Independant variables		Dependant variables OR (95% CI)				
Variables*	Categories	Physician diagnosed asthma	Attacks of shortness of breath	Any wheeze	Recurrent wheeze	Sputum production
**Family history of asthma**	Yes	2.61 (2.31-2.94)	2.44 (2.17-2.74)	2.01 (1.82-2.21)	2.35 (2.05-2.68)	1.67 (1.50-1.86)
						
**Rhinitis**	Yes	5.41 (4.81-6.08)	4.96 (4.44-5.53)	2.94 (2.69-3.21)	2.72 (2.39-3.09)	2.02 (1.83-2.22)
						
**Smoking**	Ex-smokers	1.34 (1.16-1.55)	1.29 (1.13-1.48)	1.39 (1.24-1.55)	1.35 (1.14-1.59)	1.23 (1.09-1.39)
	Smokers	1.14 (0.98-1.33)	1.81 (1.58-2.07)	3.37 (3.04-3.72)	3.88 (3.36-4.47)	2.62 (2.36-2.92)
						
**Age**	31-45	0.84 (0.72-0.98)	1.07 (0.92-1.24)	1.01 (0.90-1.14)	1.28 (1.05-1.55)	0.81 (0.71-0.92)
	46-60	0.74 (0.63-0.87)	1.06 (0.91-1.25)	1.06 (0.94-1.20)	1.71 (1.42-2.07)	0.81 (0.71-0.92)
	61-75	0.90 (0.75-1.07)	1.32 (1.12-1.56)	1.20 (1.05-1.37)	2.21 (1.82-2.69)	1.10 (0.96-1.26)
						
**Region**	Gothenburg	0.95 (0.85-1.06)	1.04 (0.93-1.16)	1.06 (0.97-1.15)	1.12 (0.99-1.27)	1.17 (1.06-1.28)
						
**Sex**	Women	1.17 (1.05-1.32)	1.46 (1.30-1.63)	1.10 (1.01-1.20)	0.91 (0.80-1.03)	0.98 (0.89-1.07)

Risks in odds ratios (OR) with 95% confidence intervals (95% CI).

* As compared to no family history of asthma, no rhinitis, non-smokers, 16-30 years of age, living in West Gothia and men respectively.

Compared with asthma, *attacks of shortness of breath *had a similar risk factor pattern with slightly lower OR for rhinitis and family history of asthma, but higher for female sex, OR 1.46 (1.30-1.63). Furthermore, increasing age was a significant risk factor for this symptom, as was both ex-smoking and current smoking (Table 4).

*Any wheeze *was less associated with rhinitis, family history of asthma and female sex than *attacks of shortness of breath*, which was similarly associated with age but yielded an OR of 3.37 (3.04-3.72) for current smoking. *Recurrent wheeze *was more age dependent and more strongly associated with current smoking, OR 3.88 (3.36-4.47) than *any wheeze*, but was not dependent on sex. Moreover, *sputum production *presented a risk factor pattern that was similar with both *any wheeze *and *recurrent wheeze *although with considerably lower odds ratios. Furthermore, living in the metropolitan area of Gothenburg was also slightly associated with *sputum production *and yielded an OR of 1.17 (1.06-1.28) (Table 4).

The most age dependent symptom was *dyspnoea*, for which age 61-75 yielded an OR of 4.60 (3.73-5.67), followed by *chronic productive cough*, OR 2.25 (1.83-2.75) and *recurrent wheeze*, OR 2.21 (1.82-2.69), both in the same age group.

## Discussion

This study presents the most updated information on the current prevalence of asthma and respiratory symptoms in northern Europe. Furthermore, the study allows for analyses of change in prevalence over eighteen years, for which there is no published recent evaluation using similar methods. Importantly, the overall message of this study is that the previously demonstrated increase in prevalence of asthma has levelled off in the region. Furthermore, most respiratory symptoms have significantly decreased in prevalence.

The prevalence of physician-diagnosed asthma in this study was estimated to be 8.3% and was greater in women than men. The current questionnaire study used nearly identical questions to the ones used in studies performed in 1996 in Finland, Estonia and Sweden (the FinEsS Studies), as well as in other studies performed in three different regions of Sweden, the capital Stockholm, the county of Norrbotten and the city of Örebro [10,24-26]. In both Örebro and Stockholm, the prevalence of asthma was estimated to be 8%, while it was 10% in Norrbotten [10,24-26]. Thus, our study together with the previous studies strongly support the notion that the prevalence of asthma in Sweden is currently between 8-10%, with minor regional variation, with no further increase observed since the late 1990s.

When comparing our results with the Gothenburg part of the ECRHS performed eighteen years before our study, the reported prevalence of asthma had increased from 6% to 8% in the same area of Gothenburg, the Hisingen Island [31]. However, this comparison must be judged with some reservation, because the difference is modest and the questions about asthma were not exactly identical. An increase of a similar magnitude was also observed in the southern part of our study area, the former county of Southern Älvsborg [32], but in this case using identical questions about asthma. Whether this increase after early 1990s reflects a real increase in asthma prevalence cannot be firmly verified, as the symptoms of asthma in the currently investigated individuals have been reduced. Thus, our findings suggest that a greater proportion of mildly symptomatic asthmatics today are diagnosed as having asthma, since the prevalence of *active asthma *was only 6.9% in the current study, and about half of these individuals used either asthma medication or had only one or two symptoms common in asthma (Figure 2). Those diagnosed with asthma in the 1980s and 1990s had clearly more symptoms of asthma than found in the current study and other recent studies [23,33]. In addition, a greater proportion of asthmatics utilised asthma medication in the 1980s and the early 1990s compared to the individuals with diagnosed asthma in our study. These two findings together argue that a greater proportion of patients with mild asthma and asthma like symptoms have received the diagnosis of asthma compared to the early 1990s. This conclusion is further supported by the studies of the incidence of asthma in northern Sweden from 1986 to 1996, which discovered that approximately half of the cases were as a result of better detection of asthma and of increased diagnostic activity within the medical community [28]. Therefore, the slight increase in doctor's diagnosis of asthma comparing our study with the two studies in 1990 and 1994 may be explained by an increased diagnosis of asthma rather than a true increase in prevalence.

In contrast to the decrease in prevalence of respiratory symptoms, our study demonstrated a clear increase in the prevalence of allergic rhinitis in the area of Hisingen compared to the 1990 ECRHS study results [31]. As allergic rhinitis is closely associated with allergic sensitisation, this marked increase might reflect an increase in allergy sensitisation in the area. While there is no recent data about the prevalence of allergic sensitisation in Sweden, ongoing clinical examinations of the current cohort will provide such information in the next few years. As rhinitis is a risk factor for the development of asthma, it cannot be excluded that the prevalence of asthma may again increase in Sweden in the future.

The argument that a plateau in asthma prevalence has been reached after the late 1990s is supported by the decrease or lack of increase of the prevalence of respiratory symptoms in different age groups in the current study. Comparing our results with the prevalence of symptoms in the 1990 ECRHS study in Gothenburg [31], almost all respiratory symptoms decreased significantly (Figure 3). Symptoms that may be related to smoking, such as *any wheeze*, *sputum production *and *longstanding cough *were reduced, but symptoms that are closely related to asthma including *wheezing with breathlessness *also decreased markedly. A clear decrease in the prevalence of wheezing was also observed in southern Älvsborg. No major changes were found regarding other respiratory symptoms, which may in part be explained by some differences in the questionnaires [32].

The decrease in prevalence of respiratory symptoms in the area of Hisingen in Gothenburg may have several explanations. Firstly, a major decrease in smoking prevalence was observed. Furthermore, a change in socio-economic status composition has been observed in parts of the area, from predominantly working class to middle class. In addition, this area contained pollution emitting industries until approximately the 1980s. Thus, changes in smoking habits, industrial structure and socio-economic status composition are all parallel with the decrease observed in respiratory symptoms. It should also be considered that the reduction in respiratory symptoms may partly be due to patients with airway diseases now having access to more efficient medications.

The demonstrated risk factor patterns for asthma and symptoms in the current study confirm findings from previous studies [23,24,26,33]. Rhinitis was strongly associated with asthma, and the magnitude of the odds ratio was similar to that previously reported in asthma studies of Swedish adults [24]. As the study design was cross-sectional, the results only verify an association and cannot contribute to the discussion of either cause or consequence. A family history of asthma was significantly related to both asthma and respiratory symptoms, but tended to be somewhat less related to asthma than found previously [24,33], a fact that may be explained by a broader labelling of the term asthma by the medical community, and the inclusion of patients with milder disease in this category. In agreement with previous studies, female sex and ex-smoking was closely associated with asthma, while current smoking was only associated with asthma with borderline significance [24,26,33].

Regarding age, the multivariate analysis verified that young adults are at highest risk of having a diagnosis of asthma. Interestingly, studies performed in the 1990s found asthma to be most common in adolescents and young adults [24], closely related to a high incidence in children and teenagers [34,35]. In the current study, the prevalence was highest in the age group of 26-35 year olds (physician-diagnosed asthma 10.2%), while it was lower in the age group of 16-25 year olds, arguing against a further increase in prevalence of asthma in the lower age group. The current findings, together with findings from several studies presented in the last decades, argue that the continued increase in asthma prevalence that has been observed over half a century is now over.

All respiratory symptoms were significantly associated with smoking. The symptoms most closely related to smoking were any and recurrent wheeze, cough and sputum production. These findings are similar to reports from several Scandinavian studies [23,26,36]. Chronic respiratory symptoms increased with increasing age, a relationship that was confirmed by the multivariate analysis. However, it was observed that symptoms were less age-dependent compared with previous Swedish studies [21,23,27]. High age (61-75 years) was poorly related to most respiratory symptoms with odds ratios of 1.00 to 1.32, and most of these symptoms did not significantly relate to age.

The differences in prevalence in asthma and respiratory symptoms between the metropolitan area of Gothenburg and the non-metropolitan area were strikingly small. All asthma-associated variables were equally common in the two samples, while the prevalence of most symptoms was only slightly, but significantly, more common in the city of Gothenburg. Thus, in the multivariate analyses, the area of domicile, i.e. living in Gothenburg, was a significant risk factor only for sputum production. These results may reflect an improvement in the levels of outdoor air pollution in the metropolitan areas in Sweden [37].

This study provides conclusive results for several reasons. The large sample size and the use of well validated questionnaires contribute to high internal validity of the results. As identical questions have been used in several previous studies, many opportunities for comparisons were available, and can contribute to further analyses. Therefore, the external validity can be judged as high, partly because this study can be utilised for future comparisons. The response rate was slightly lower than in earlier Swedish and Nordic studies [23-29]. However, in a study of non-responders, no important bias was detected between early and late responders [30]. The alternative of answering over the internet was utilised by surprisingly few individuals, but could still be a more efficient way of working in the future, especially with younger generations. Postal questionnaires always have one key weakness in that they can never provide evidence for any direct causal reasons or mechanistic information in any disease. Furthermore, the cross-sectional design itself makes discussion of cause or consequence, as well as detected associations, less convincing.

In conclusion, our study provides new and unique evidence that the previous increase in asthma prevalence over the last 10-15 years in West Sweden has now levelled off. Asthma is still highly prevalent, with 8.3% of the population being affected, which makes it one of the most common diseases in Sweden. Furthermore, asthma can still be lethal, and the incidence of asthma mortality in children and young adults has only partly decreased in the last decade [38].

## Competing interests

The authors declare that they have no competing interests.

## Authors' contributions

JL conceived of the study, participated in its design and drafted the manuscript. LE participated in the collection of data, preformed the statistical analysis and helped draft the manuscript. EPR revised the manuscript. GW conceived of the study, participated in its design and revised the manuscript. AL conceived of the study, participated in its design and revised the manuscript. ER conceived of the study, participated in its design and revised the manuscript. KT conceived of the study, participated in its design and revised the manuscript. BL conceived of the study, participated in its design, supervised the analyses and drafted the manuscript. All authors read and approved the final manuscript.
